# Reply to “Describing center of pressure movement in stabilometry by ellipse area approximation” from Agnieszka Gołąb concerning the paper “A Review of Center of Pressure (COP) Variables to Quantify Standing Balance in Elderly People: Algorithms and Open Access Code”

**DOI:** 10.14814/phy2.15416

**Published:** 2022-08-26

**Authors:** Flavien Quijoux, Alice Nicolaï

**Affiliations:** ^1^ ORPEA S.A. Research Department Puteaux France; ^2^ Centre Borelli UMR 9010/Université Paris‐Saclay, ENS Paris‐Saclay, CNRS, SSA, Inserm Université Paris Cité Paris France

## Abstract

Letter to the Editor concerning “Describing center of pressure movement in stabilometry by ellipse area approximation” from Agnieszka Gołąb.

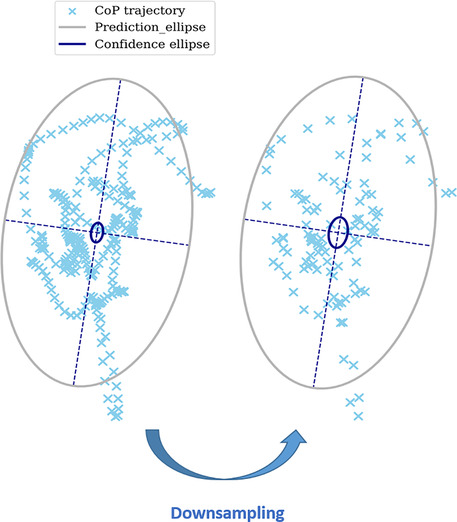

Dear Editors,

We thank Agnieszka Golab for the opportunity to bring precisions and additional definitions and formulas concerning the features used in stabilometry that are based on ellipse area calculations. Our choice was actually to present the formula of the prediction ellipse area in the article, as it indeed does not strongly depend on the sample size as the confidence ellipse area does. However, we first created a undesirable confusion by choosing the name “confidence ellipse area”—which is the most common name used in posturography regardless of the method actually used. Secondly, the definition at the beginning of the paragraph does not correspond to the formula that is provided, the later is the one used in the articles results and available in the open‐access code. Therefore, we agree that a clarification is required and take the opportunity of this letter to present rigorously the two different definitions of the confidence ellipse area and the prediction ellipse area. The proofs of these formulas are given in (Schubert & Kirchner, 2014). The later article also cites the different variations of these features that have been proposed such as the standard ellipse area which is a special case of the prediction ellipse area (Schubert & Kirchner, 2014). The confidence ellipse area is defined as the area of the ellipse that contains the true mean of the signal with a probability of 95%. As sample size increases, the confidence area will decrease because the location of the true mean is known with less uncertainty. This is reflected in the formula by the presence of the coefficient 1/N.

$$\begin{array}{c} \text{95\%}-\text{Confidence ellipse area}=2\pi \times \frac{1}{N}\times \frac{N-1}{N-2}\times F_{0.95,2,N-2}\\ \;\times \sqrt{\sigma_{\mathrm{ML}}^{2}\sigma_{\mathrm{AP}}^{2}-\sigma_{\mathrm{ML}-\mathrm{AP}}^{2}}\;, \end{array}$$

where *N* is the length of the signal, *σ*_ML_ and *σ*_AP_ are the standard deviations of the mediolateral and anteroposterior time series, respectively, and *σ*_ML−AP_ is the covariance of the ML and AP time series.

The prediction ellipse area, which is the feature computed in the open‐access code proposed in (Quijoux et al., 2021), is defined as the following:

$$\begin{array}{c} \text{95\%}-\text{Prediction ellipse area}=2\pi \times \frac{N+1}{N}\times \frac{N-1}{N-2}\times F_{0.95,2,N-2}\\ \;\times \sqrt{\sigma_{\mathrm{ML}}^{2}\sigma_{\mathrm{AP}}^{2}-\sigma_{\mathrm{ML}-\mathrm{AP}}^{2}}\;. \end{array}$$

We agree with Agnieszka Golab that the prediction ellipse area formula, which shows only marginal differences with variations in sample size (Schubert & Kirchner, 2014), should be used instead of the confidence ellipse area. We also agree that it is relevant to present the method to calculate the orientation angle of the ellipse according to the chosen axis (mediolateral or anteroposterior). Although the calculation consists of deducting 90° from one of the angles to obtain the value for the other axis, this precision is not always provided, which limits the standardization of this feature. This review is indeed intended to be continued with the implementation of other features relevant for the prediction of fall risk in the elderly. Entropy variables (Ramdani et al., 2013) or wavelet analyses (Mart'ınez‐Ram'ırez et al., 2011) may be relevant in the future, but are beyond the scope of this review. This compendium is, as pointed out by Agnieszka Golab, a starting point toward more standardization in the presentation of features used in the study of posture, and thus is not meant to be exhaustive.
